# Association between spontaneous peer support experience and psychosocial factors among heart transplant recipients

**DOI:** 10.3389/fcvm.2026.1888455

**Published:** 2026-07-27

**Authors:** Alexandra Assabiny, Orsolya Papp-Zipernovszky, Zsófia Ocsovszky, Blanka Ehrenberger, Béla Merkely, Beáta Dávid

**Affiliations:** 1 Heart and Vascular Centre, Faculty of Medicine, Semmelweis University, Budapest, Hungary; 2 Doctoral School of Mental Health Sciences, Semmelweis University, Budapest, Hungary; 3 Department of Personality and Health Psychology, Psychological Institute, Faculty of Education and Psychology, Eötvös Loránd University, Budapest, Hungary; 4 Doctoral School of Cardiovascular Medicine and Research Program, Semmelweis University, Budapest, Hungary; 5 Institute of Mental Health, Semmelweis University, Budapest, Hungary

**Keywords:** heart transplantation, medication adherence, peer support, psychosocial factors, quality of life

## Abstract

**Introduction:**

Peer support in heart transplantation (HTX) remains underexplored.

**Methods:**

We investigated recipients' experiences with peer support and its associations with quality of life, health literacy (BRIEF), medication adherence, depressive symptoms (BDI-H) and alcohol consumption (AUDIT).

**Results:**

Participants (*n* = 201, 152 male) evaluated whether they received peer support across four phases of transplant journey: pre-waiting list, waiting list, intensive care unit and first post-transplant year (FY). Support was rated as positive, negative, not received but requested, or not received and not requested. Peer support was significantly more frequent after transplantation than before (75% vs. 48%, *p* < 0.001). A notable proportion did not receive support despite wishing for it (19.9 -7%). Most participants reported peer support as highly beneficial, and no negative experiences were reported. No psychosocial differences were observed between supported and unsupported recipients pre-HTX.Those receiving support in the FY had higher education attainment. Individuals who would have requested support showed the lowest BRIEF, the highest BDI-H and AUDIT scores. Regression analyses revealed no predictive effects of peer support.

**Discussion:**

Overall, peer support is common and positively perceived among HTX recipients. However, individuals with unmet support needs during the first post-transplant year may represent a vulnerable subgroup, underscoring the need for more structured peer support interventions.

## 1 Introduction

Adaptation to lifestyle changes, medication regimens, and emotional distress are among the various physical and psychological challenges faced by heart transplant (HTX) recipients (1). Compared with the general population, heart transplant recipients exhibited substantially higher prevalence rates of depression (21.6% vs. 12.9%), anxiety (11.1% vs. 7.3%), and post-traumatic stress disorder (13.5% vs. 3.9%) (2). Beyond these psychiatric disorders, qualitative studies have identified several psychologically significant experiences across the heart transplantation journey. Experiences in the intensive care unit may be profoundly distressing, including pain, delirium, hallucinations and social isolation (3). Furthermore, kinesiophobia may negatively affect postoperative physical rehabilitation (4), while donor and donation images (DDIs)—defined as “all (magical and/or rational) thoughts and feelings that an organ recipient has associated with the donor or the donated organ itself"—may be associated with feelings of guilt and difficulties in accepting the transplanted organ (5).

As a relevant psychosocial factor, social support contributes to post-transplant adherence, overall well-being, and prevention of substance use relapse (6, 7). It is also associated with better outcomes during the pretransplantation wait, such as longer survival and less reliance on mechanical circulatory support (8). As a multifaceted construct, social support encompasses emotional, instrumental, and informational support provided by various sources, including family, friends, peers, the community, and other personal networks (9). Measuring social support is challenging and requires several methodologies, potentially yielding inconsistent results (10). Therefore, transplantation guidelines and consensus documents do not accurately define social support (11, 12). Existing psychosocial evaluations before transplantation systematically assess the availability, stability, and adequacy of available support resources and provide caregivers with essential information regarding the patient's treatment and care needs (12). In addition to the source of support, its timing might be also decisive during HTX.

Despite the uniqueness of the HTX experience, the peer dimension of social support is relatively understudied. Only peers can understand the transplantation experience, which forms a crucial aspect of their emotional support (13). Peer information provision can be as important as that provided by the medical personnel; the latter is essential for successful post-transplant outcomes. However, information gaps remain major challenges for many patients, necessitating further education and innovative support networks (13–16).

There have been a limited number of studies that focused on peer support among adult HTX populations, using qualitative methods with relatively small sample sizes (17). Semi-structured interviews have been used in qualitative studies. Mauthner et al. (13) found that 40% of the HTX recipients referred to peers as the best form of support, despite the fact that 40% of those recipients had never received peer support. Fatma et al. (18) found that more than half of the participants (*N* = 11) spontaneously mentioned emotional connections with their peers during the interviews. The authors recommended further quantitative studies with larger numbers of participants. The only quantitative evaluation of a local peer mentoring program published by Wright et al. (19) involved 16 participants. The authors reported that the ratings of the usefulness of peer mentoring support were consistently high in the informational, emotional, and coping domains. The main topics of peer discussions were medical issues, such as postoperative complications, medications, waiting for transplantation, and surgery. Emotional issues and personal topics, such as sexual relations and marital problems, were less frequent. The latest publication on peer support in the heart transplant population have primarly focused on structured peer mentor programs targeting pediatric and young adult population and/or virtual interventions. Jerson et al. (20) demonstrated that adolescent transplant recipients acting as peer mentors improved their self-management skills and health-related quality of life. Criss et al. (21) evaluated a virtual pediatric transplant peer mentoring program, finding it acceptable and useful in reducing isolation and fostering a supportive community among participants. In addition, Anthony et al. published a series of studies (22–24) describing how qualitative findings informed the development and design of a peer support program.

The aim of our study is to assess HTX recipients’ experiences with spontaneous peer support and its relationship to health-related psychological status using quantitative methods. The findings of this study will address the knowledge gap in the literature and provide deeper insights into the longer-term effects of peer support in this patient population.

## 2 Materials and methods

Our cross-sectional quantitative study was approved by the ethics committee of Semmelweis University Regional and Institutional Committee of Science and Research Ethics (Nr. SE RKEB 166/2023) and was conducted in accordance with the principles of the Declaration of Helsinki. The participants were recruited consecutively during routine follow-up visits at the Semmelweis University Heart and Vascular Centre between 2023 and 2025. Before and during the study period, no organised peer support program was available for recipients.

### 2.1 Participants

We enrolled 212 adult recipients of HTX at the Semmelweis University Heart and Vascular Center. The eligibility criteria were HTX more than 3 months prior, and signed informed consent. The exclusion criteria were the presence of physical limitations in completing the questionnaire and refusal to provide informed consent (*n* = 11).

### 2.2 Measurements

The questionnaire included sociodemographic variables, such as age, marital status, and years of education (Table 2).

#### 2.2.1 Peer support experience

Patients retrospectively evaluated their experience of peer support during transplantation by dividing it into the following four periods: (1) before the waiting list (preWL), (2) during the waiting list (WL), (3) during the intensive care unit period (ICU), and (4) in the first year post-transplantation (FY). PreHTX (preWL, WL) and postHTX (ICU, FY) groups from the four stages were created, where applicable.

The impact of peer support on the transplantation process was assessed by participants using the following response categories: (a) indifferent, (b) small positive extent, (c) medium positive extent, (d) large positive extent, (e) negative, (f) did not receive peer support but would have requested it, and (g) did not receive peer support but had not requested it.

During the analyses, we made two comparisons between the derived groups out of the six evaluation possibilities to retrospectively identify the need and availability of peer support (Table 1).

**Table 1:** Comparison groups to identify the experience with peer support .

Quality of peer support experience	Comparison1	Comparison2
Received, negatively affected the process	RECEIVED1	RECEIVED2
Received, indifferent		
Received, small positive extent		
Received, moderate positive extent		
Received, large positive extent		
Did not receive peer support, but would have requested for it	NOT RECEIVED1	WOULD HAVE REQUESTED2
Did not receive peer support, had not requested for it		NOT REQUESTED2

#### 2.2.2 Behavioral, and psychosocial factors

Quality of life was measured using the visual analogue scale (EQ VAS) from the EQ5D (25), which assesses the patient’s self-rated health on a vertical scale, with two endpoints: ranging from 0 to 100, from ‘the worst health’ to ‘the best health you can imagine’.

Health literacy was assessed using brief health literacy screen (BRIEF) (26, 27), a 4-item screening measurement that assesses patients’ self-perceived difficulties in understanding and handling medical forms, and verbal and written information about their health conditions. Higher scores indicated higher levels of health literacy. The Hungarian version is reliable (Cronbach alpha = 0.87), however, in our sample, all four items had low reliability (Cronbach's alpha = 0.562). Therefore, based on further item-total statistics, we used only the first three questions as the deletion of the item ‘How confident are you in filling out medical forms by yourself?’ indicated acceptable reliability (Cronbach's alpha = 0.795). Our decision is also supported by both the original and the Hungarian validation of BRIEF: this is a reversed and differently scored item than the other three. Furthermore, in the Hungarian validation, this item showed the lowest item-total correlation with the other items (0.53), and a slight improvement could have been achieved after deleting it.

We used the 5-item Medication Adherence Report Scale (MARS-5) (28), adapted for immunosuppressive medication (MARS-5i), to assess chronic patients’ adherence behaviour and decisions related to medication. The tool uses a 5-point Likert scale, with the summed scores ranging between 5 and 25; a higher score indicates better adherence. The Cronbach's alpha for our sample was 0.814.

The 9-item Beck Depression Inventory (BDI-H) (29) measures the severity of mood disorders by asking patients to agree on indecision, physical symptoms (e.g., fatigue and sleep disturbance), social withdrawal, incapacity to work, self-blame, and lack of joy on a 4-point Likert scale. This shortened version showed an extremely high correlation with the 21-item Beck Depression Inventory (r = .92) and is widely used in Hungarian studies to screen different clinical populations (29, 30) as well as for convergent validation purposes [e.g., 5-item version of the WHO Well-Being Index (WBI-5) (31)]. Higher total scores indicate more severe depressive symptoms. The 9-item BDI-H showed a good internal reliability in our sample (Cronbach alpha = 0.827). We use it solely as a screening measure rather than as a comprehensive psychological assessment.

Alcohol-related problems were screened using The Alcohol Use Disorders Identification Test (AUDIT) (32), which consists of 10 items. Higher scores indicate more problematic alcohol consumption. Item 9 (‘Have you or someone else been injured because of your drinking?’) was excluded from the reliability analysis because of a lack of variability in the answers. The Cronbach's alpha in our sample was 0.732, which is acceptable.

### 2.3 Statistical analysis

Statistical analyses were performed using IBM SPSS Statistics for Windows version 27. According to the central limit theorem, the sample size of this study allowed us to conduct parametric statistical trials (33). We used related-samples Cochrane Q-test to test whether the distributions show significant differences during the periods of HTX. To assess the differences in continuous variables between those who received peer support and those who did not, we conducted group comparisons: independent sample *t*-tests in the case of Comparison1 (Table 1) and one-way Anova in the case of Comparison2 (Table 1). The Hochberg T2 *post-hoc* test was used when the criteria for homogeneity of variances were met; otherwise, the Dunnett's T3 test was applied. For categorical variables (e.g., sex), chi-square tests were used to determine whether the distributions showed significant differences during the HTX process. To test the longer-term effects of peer support experience linear regression analyses were performed. The level of significance was set at *p* = 0.05.

## 3 Results

A total of 201 adult HTX patients were included in the analysis. The baseline characteristics of the patients are listed in Table 2.

**Table 2:** Baseline characteristics.

Variables	N (%)
Sex	
Male	152 (75.6)
Female	49 (24.4)
*Age, years*	56.3 ± 11.5 (min. 19, max. 80)
*Time since transplant, years*	6.4 ± 4.0 (min. 0, max. 27)
*Years spent in education, years*	13.3 ± 3.3 (min. 3, max. 25).
*Marital status*	N (%)
married or in a relationship	142 (70.64)
not having a partner	58 (28.86)
Missing	1 (0.5)
*Health related variables*	Mean ± SD
EQ VAS (%)	80.2 ± 17.0 (min. 12, max. 100)
BRIEF (3 item)	9.6 ± 2.2 (min. 2, max. 12)
MARS-5i	24.6 ± 1.2 (min. 14, max. 25)
BDI-H	2.9 ± 3.3 (min. 0, max. 19)
AUDIT	1.7 ± 2.6 (min. 0, max. 15)

### 3.1 Peer support experience

**Figure 1:**
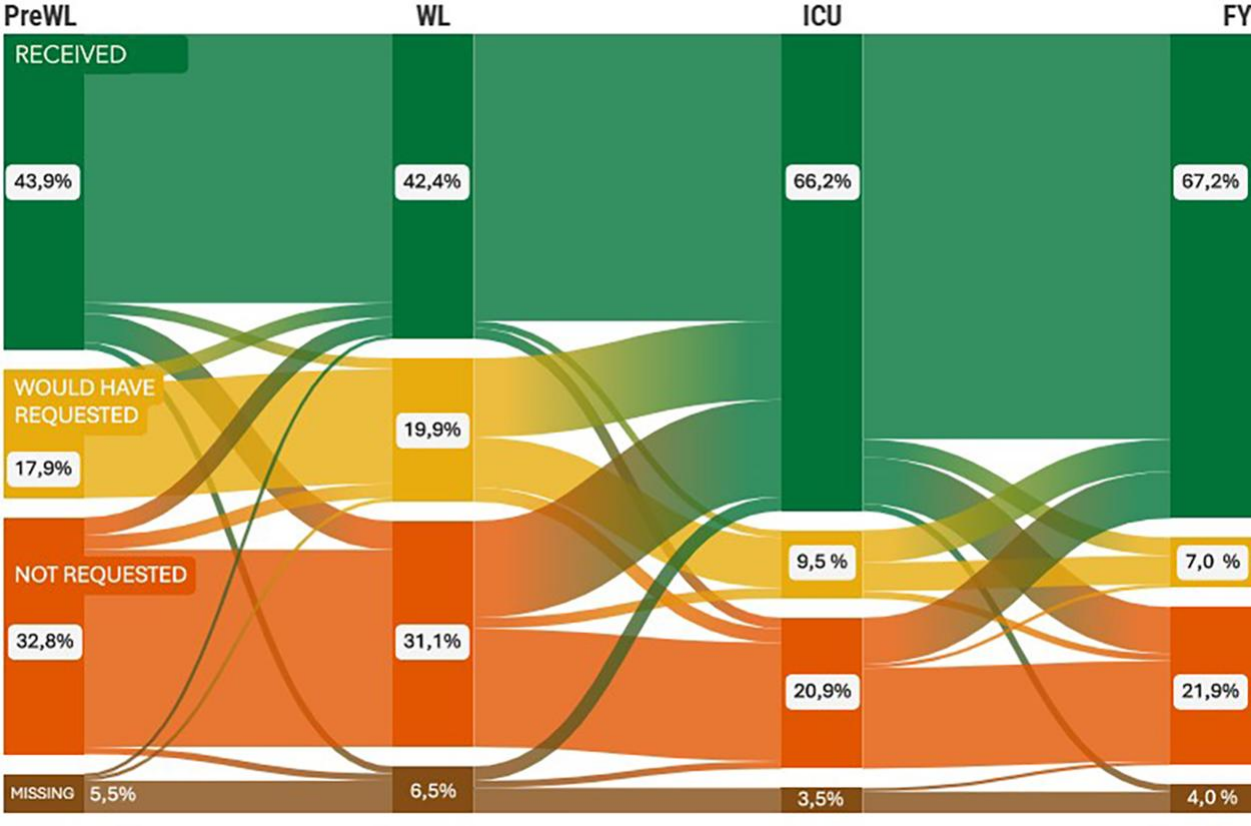
Sankey diagram visualising the distribution and transitions of three quality groups across four periods (before the waiting list (preWL), during the waiting list (WL), during the intensive care unit care (ICU), and during the first post-transplant year (FY)). Each vertical column represents the experience of the population at a specific period; the flows between the columns indicate the transitions of individuals between groups over time.

We visualised spontaneous peer support experience journey in Figure 1. Significantly more patients received peer support during the post- than pretransplant periods [pre-HTX (48%) vs. postHTX (75%); Chi^2^ = 52.536, *p* < 0.001]. Across four phases, the lack of peer support was more frequently attributed to not requesting peer support than to requesting but not receiving it (pre-WL 32.8% vs. 17.9%; WL, 31.3% vs. 19.9%; ICU, 20.9% vs. 9.5%; FY, 21.9% vs. 7.0%).

#### 3.1.1 Quality of peer support experience

**Figure 2:**
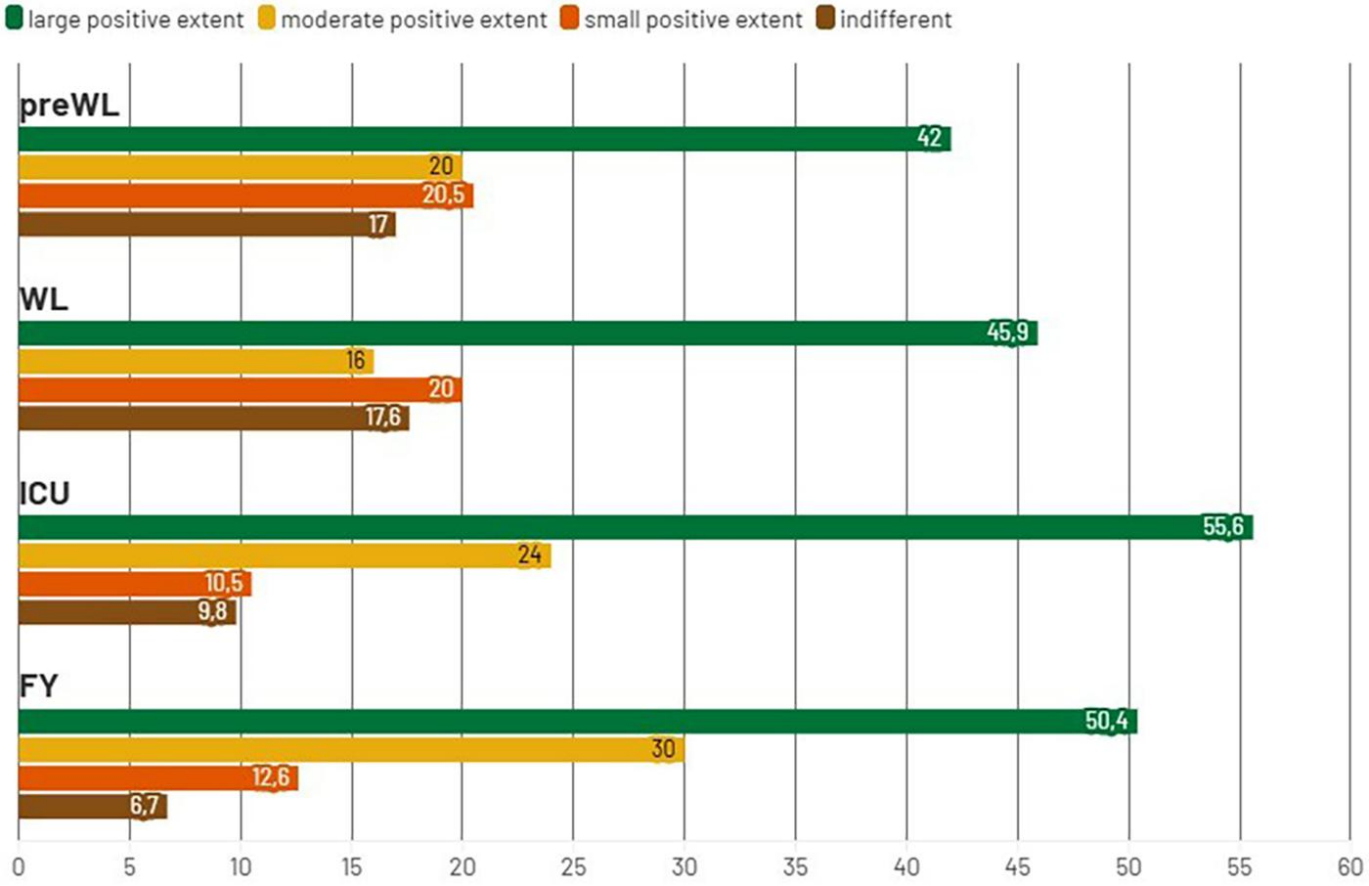
Percent distribution of the received support quality by time. The impact of peer support on the transplantation process during the four periods (before the waiting list (preWL), during the waiting list (WL), during the intensive care unit care (ICU), and during the first post-transplant year (FY) assessed using the following response categories: indifferent, small positive extent, medium positive extent, large positive extent, negative.

We assessed the quality of the support received over time (Figure 2). Most respondents evaluated this support as helpful to a large positive extent during all stages of the HTX process. In our population, none of the participants experienced negative peer support.

#### 3.1.2 Consistent peer support experience journey

71.1% of the respondents’ experience depended of the phases, only 28.9% of the respondents evaluated their experience quality consistently during the four phases (Table 3). The most frequent consistent evaluations were not requested peer support (11.4%) and large positive effect (10.4%).

**Table 3:** Consistent evaluation of peer support.

Peer support experience	Number of consistent experience, *n* (%)
large positive extent	21 (10%)
medium positive extent	4 (2.0%)
small positive extent	1 (0.5%)
indifferent	5 (2.5%)
would have requested	4 (2.0%)
not requested	23 (11.4%)
sum consistent experience	58 (28.9%)

### 3.2 Relationship between peer support experience, behavioral and psychosocial factors

To uncover the relationship between peer support and quality of life, health literacy, adherence, depressive symptoms and alcohol consumption during the four periods of transplantation we conducted further group (Table 1) analyses.

During the pre-transplant period, no significant differences were observed between the groups in our variables (Supplement 1), whereas the post-transplant groups significantly differed, which is detailed in the following sections.

#### 3.2.1 Education

In the group comparisons, the level of education showed a significant difference across the levels of perceived peer support (Table 4, Supplement 2, 3).

**Table 4:** Significant results in health-related variables: comparisons of evaluating peer support among the groups during the posttransplant period.

Variables	Comparison1.				Comparison2.					
	RECIEVED1	NOT RECEIVED1	Sig	F/chi (df)	RECEIVED2	WOULD HAVE REQUESTED2	NOT REQUESTED2	Sig	F/chi (df)	posthoc
ICU period										
Education in years	13.42 (3.60)	13.038 (2.74)	0.458	−0.744 (130,82)	13.415 (3.60)	12.588 (2.15)	13.250 (2.98)	0.637	0.452 (2)	
MARS-5	24.59 (0.95)	24.54 (1.55)	0.788	−0.269 (175)	24.59 (0.95)	24 (2.56)	24.80 (0.56)	0.049*	3,065 (2)	NS
BRIEF (3 item)	9.56 (2.09)	9.64 (2.57)	0.815	0.234 (187)	9.56 (2.09)	8.68 (3.48)	10.10 (1.88)	0.074	2.645 (2)	
BDI-H	2.67 (2.72)	3.47 (4.31)	0.192	1.315 (80,71)	2.67 (2.72)	5.56 (5.87)	2.56 (3.08)	0.002 *	6.618 (2)	NS
AUDIT	1.56 (2.24)	2.02 (3.23)	0.307	1.025 (154)	1.56 (2.24)	3.07 (4.20)	1.50 (2.54)	0.09	2.444 (2)	
First year period										
Education in years	13.778 (3.52)	12.24 (2.61)	0.002*	−3,116 (123,97)	13.778 (3.52)	11.923 (2.29)	12.351 (2.73)	0.022*	3.912 (2)	S
MARS-5	24.60 (0.89)	24.52 (1.65)	0.656	−0.447 (175)	24.60 (0.89)	23.93 (2.97)	24.71 (0.84)	0.088	2.467 (2)	
BRIEF (3 item)	9.72 (2.06)	9.32 (2.65)	0.262	−1.02 (86,75)	9.72 (2.06)	7.79 (3.17)	9.81 (2.28)	0.007*	5.128 (2)	S
BDI-H	2.66 (2.84)	3.56 (4.25)	0.159	1.422 (73,53)	1.66 (2.49)	6.57 (6.01)	2.50 (2.86)	<0.001**	10.023 (2)	NS
AUDIT	1.66 (2.49)	1.78 (2.77)	0.798	0.256 (154)	1.66 (2.49)	4.38 (5.26)	1.15 (1.20)	0.005*	5.435 (2)	NS

S, significant; NS, not significant.* <0.05.** <0.01.

In Comparison 1, ‘RECEIVED1’ vs. ‘NOT RECEIVED1’ groups education significantly differed in FY [t(123.970)= −3.116, *p* = 0.006] and during the post-HTX period [t(71.735)= −2.569, *p* = 0.012]. Those who spent more years in education received peer support.

During Comparison 2 ‘RECEIVED2’ vs. ‘WOULD HAVE REQUESTED2’ vs. ‘NOT REQUESTED2’ groups, spending more years in education also made a significant difference [F(2) = 3.912, *p* = 0.022] in FY experience: the ‘RECEIVED2’ group spent significantly more years in education than the other two groups.

#### 3.2.2 Health literacy

The group comparisons also revealed significant differences based on health literacy scores. (Table 4, Supplement 2, 3) No significant differences were found in Comparison 1 (‘RECEIVED1’ vs. ‘NOT RECEIVED1’). In Comparison 2, higher health literacy showed a significant difference [F(2)= 5.128, *p* = 0.007] in FY experience: the ‘WOULD HAVE REQUESTED2’ group exhibited the lowest health literacy, differing from the other two groups.

#### 3.2.3 Alcohol consumption and depressive symptoms

We found a similar pattern in the case of the AUDIT and 9-item BDI-H; although the initial analyses indicated significant differences between the groups, they did not remain so in the *post hoc* analyses. (Table 4, Supplement 2, 3).

Significant differences in the mean score of depressive symptoms were observed among the groups during both the FY [F(2)= 10.023, *p* < 0.001] and ICU periods [F(2)= 6.618, *p* = 0.002]; however, post hoc analyses did not reveal significant pairwise differences. In both cases, the highest depressive symptom rates were found in the respective ‘WOULD HAVE REQUESTED’ groups. Significant differences in AUDIT scores were found among peer support groups during the FY experience [F(2)= 5.435, *p* = 0.005], with the highest scores observed in the respective ‘WOULD HAVE REQUESTED’ group; however, *post hoc* analyses did not reveal significant pairwise differences.

#### 3.2.4 Quality of life and adherence

We found no significant differences for the quality of life or adherence to the immunosuppressant regimen during the HTX periods. (Table 4, Supplement 2, 3).

### 3.3 Regression analyses for long term predictions

We conducted linear regression analyses to identify possible longer-term effects of the peer support experience on quality of life, health literacy, adherence, depressive symptom and alcohol consumption scores as dependent variables. We ran these trials in individual models, where our independent variables were ‘RECEIVED1’ vs. ‘NOT RECEIVED1’; ‘RECEIVED2’ vs. ‘WOULD HAVE REQUESTED2’ vs. ‘NOT REQUESTED2’ during the transplantation journey. We found no significant predictions, only one on a tendency level: those who received peer support within the first year after transplantation have lower depressive symptom score [R^2^ = 0.015, F(181) = 2.783, *p* = 0.097].

## 4 Discussion

Early research has conceptualised ’social health’ demonstrating that social networks and ties (e.g., marital status, family, close friends, and affiliation with religious or voluntary associations) predict mortality (34). More than two decades ago, Grey et al. (35) highlighted a noteworthy paradox: although the previous decade had witnessed a significant expansion in the number of cancer peer support groups, despite this notable trend, the topic had received little systematic scientific attention within the academic literature. A similar phenomenon can be observed in heart transplantation field nowadays: while in 2020 more than 7,000 heart transplants were performed globally and social support is crucial during the heart transplantation journey, available data are primarily on caregivers, family, and friends rather than peer interactions (11, 12, 36). Herein, we evaluated the experiences of 201 adult HTX recipients with spontaneous peer support. Along with assessing the presence of spontaneous peer support, we emphasised the importance of evaluating the experiences and psychosocial status of individuals who did not receive such support.

Our findings indicate that approximately half of the patients received spontaneous peer support before heart transplantation, which increased to three-quarters post-transplantation. Most recipients perceived this support as highly beneficial, in our quantitative study none of our participants reported negative peer support experiences overall. These quantitative results align with the primary narrative data, indicating that peer support is important throughout the HTX journey (13–16, 18, 19). Indirect evidence further supports its importance; indeed, one study on an internet-based intervention found that integrated peer discussion groups were the most frequently utilised and highly rated component of the psychosocial intervention, with engagement correlating positively with psychosocial benefits (37). Our data suggest that significantly more peer connections occurred post-transplant. Centralised nature of specialised posttransplant care, preoperative coping mechanisms and personality characteristics such as avoidance or optimism also might have contributed to the reduced need for preoperative peer support (38, 39)..

To the best of our knowledge, no quantitative research has examined the relationship between spontaneous peer support, behavioral and psychosocial factors in HTX patients. To address this gap, we conducted subgroup analyses and found no significant differences between patients who received peer support and those who did not in terms of quality of life, depressive symptoms, alcohol consumption, or health literacy. Ladin et al. (10) findings related to social support and post-transplantation outcomes are inconsistent, reflecting its important, but non-directional role, which may also be true for peer connections based on our results. It is also important to point out, that our study was not designed to evaluate the long-term effectiveness of peer support. We investigated mainly those behavioral and psychosocial factors such as nonadherence, depressive symptoms and excessive alcohol consumption, which are well-known psychosocial predictors of heart transplant outcomes (11), but in this context several pathways as well as confounders may be existing. Peer support studies generally use quality of life, self-efficacy, clinical surrogates, depression, distress or health knowledge as an outcome variable, in order to deeper understand the mechanisms of planned, structured and designed peer interventions for participants (40). In this way peer support interventions affect several important domains of chronic disease management for example self-care management, empowerment or knowledge about the disease (41). In contrast, we investigated spontaneously generated experience between heart transplant recipients, additionally, we measured perceived rather than received support, which weakly correlate (r = 0.1–0.3) (42).

The basic socioeconomic variable of educational status differed significantly between patients who received posttransplant peer support and those who did not. International evidence demonstrates that higher levels of education enhance individuals’ capacities to maintain health, navigate and utilize health services (43). Consistent with this literature, our data indicate that more highly educated heart transplant recipients more frequently utilize the opportunities offered through peer-support networks.

Systematic reviews have indicated that the overall effectiveness of peer support interventions for chronic conditions remains inconclusive. Thompson et al (40). in their systematic review of reviews showed that, there are methodological weaknesses across the international literature. Furthermore, there is a lack of consistent statistically significant effects of peer support across the investigated reviews. At the same time the authors discussed their results as “at present it is not possible to draw definitive conclusions about effectiveness, although the potential for peer support interventions is apparent”. (40, p.9) Our descriptive results support the obvious importance of peer support in heart transplant population. Furthermore, the underlying needs and mechanisms of peer support have been previously explained using various theoretical frameworks, including social cognitive theory, appraisal and social support theories, social comparison theory, illness and social identity theory, the theory of planned behaviour, and empowerment theory (40). At the same time there is lack of data about those, who consciously withdrawing from peer support or not seeking it. Previous studies measuring the effectiveness of peer support interventions in heart transplant population have not examined the characteristics of recipients who do not participate in formal peer interventions, although they have provided quantitative data on effectiveness (19). Our findings indicated that 20%–30% of patients did not perceive or seek peer support at different phases of the transplant process. A more detailed subgroup analyses identified a further potentially more vulnerable group. Patients who did not receive but would have desired support within the first posttransplant year exhibited significantly lower health literacy and educational attainment and had the highest depressive symptom and alcohol consumption scores. Further qualitative investigation is needed in the future to design effective psychosocial interventional framework for this subgroup.

In summary, within the rapidly evolving healthcare landscape, peer support has emerged as an important component of high-quality care delivery, demonstrating an association with reduced overall healthcare expenditures (44, 45). Our findings are important for understanding our patient population and for intentionally designing peer-support interventions that are patient-centered and tailored to individuals undergoing heart transplantation. This perspective may also be relevant for healthcare professionals who provide medical care throughout the unique trajectory of the heart-transplantation journey.

The primary limitation of our study is that the experiences related to peer support were measured retrospectively, making methodological bias unavoidable. Additionally, owing to its cross-sectional design, the time elapsed since transplantation widely varied, distorting the assessment of past support experiences. The number of participants in the comparisons was not equal across the groups, reflecting the natural transition of information flow during the HTX process, but this also led to a decrease in the number of the ‘WOULD HAVE REQUESTED’ group. In addition, the COVID pandemic and its effect on peer relationships during the transplantation process might have impacted our results. Additional clinical characteristics, such as indication for transplantation, previous LVAD support, retransplantation status, major post-transplant complications would improve the impact of the study.

Our findings illustrate the role of spontaneous peer relationships in patients undergoing HTX. Approximately half of the patients received peer support before and three-quarters after the procedure, with most finding it highly beneficial and no reports of harm, indicating its safety. While no significant differences were found between the “received” and “not received” groups in terms of quality of life, depressive symptoms, alcohol consumption habits, and health literacy, more sophisticated subgroup analyses provide deeper insights into the importance of this dimension of social support. Herein, we identified a potentially vulnerable subgroup with a tendency towards less favourable behavioral and psychosocial outcomes. Those who did not receive but wished for support post-transplantation showed lower health literacy and education, as well as higher depressive symptom and alcohol consumption scores, highlighting the unmet needs in this population.

## Data Availability

The original contributions presented in the study are included in the article/Supplementary Material, further inquiries can be directed to the corresponding author.
