# Supplementary material for: Association between spontaneous peer support experience and psychosocial factors among heart transplant recipients
Source: Front Cardiovasc Med. 2026 Jul 27;13:1888455. doi: 10.3389/fcvm.2026.1888455 (PMC13455501; doi:10.3389/fcvm.2026.1888455)
Supplement: Supplementary file 1 [file Table1.docx]

|  | Comparsion1 | | | | Comparsion2 | | | | |
| --- | --- | --- | --- | --- | --- | --- | --- | --- | --- |
|  | RECIEVED1 | NOT RECEIVED1 | Sig | F/khi (df) | RECEIVED2 | WOULD  HAVE REQUESTED2 | NOT REQUESTED2 | Sig | F/khi (df) |
| **preWL** |  |  |  |  |  |  |  |  |  |
| Age | 55.91  (12.596) | 56.35 (10.608) | 0.79 | 0.264 (188) | 55,91 (12.596) | 56.97  (10.252) | 56.02  (10.859) | 0.892 | 0.114 (2) |
| Sex  (f/m) | 19/69 | 25/77 | 0.634 | 0,226 (1) | 19/69 | 11/25 | 14/52 | 0.504 | 1.369 (2) |
| Education in years | 13.692  (3.359) | 12.911  (3.235) | 0.134 | -1.506 (161) | 13.692 (3.359) | 12.943  (3.85) | 12.891  (2.813) | 0.326 | 1.130 (2) |
| EQ VAS | 79.67 (17.853) | 80.60  (16.766) | 0.713 | 0.368 (185) | 79.67 (17.853) | 83.03 (15.242) | 79.29 (17.505) | 0.55 | 0.600 (2) |
| MARS-5i | 24.51  (1.041) | 24.62  (1.306) | 0.554 | 0.593 (171) | 24.51  (1.041) | 24.44  (1.910) | 24.72  (0.799) | 0.461 | 0.778 (2) |
| BRIEF  (3 item) | 9.76  (1.988) | 9.51  (2.347) | 0.441 | -0.771 (183) | 9.76  (1.988) | 8.97  (2.767) | 9.78  (2.073) | 0.162 | 1.839 (2) |
| BDI-9 | 2.80  (2.943) | 3.03  (3.674) | 0.644 | 0.463 (178) | 2.80  (2.943) | 3.54  (3.616) | 2.73  (3.705) | 0.472 | 0.754 (2) |
| AUDIT | 1.81  (2.591) | 1.60  (2.583) | 0.62 | -0.497 (151) | 1.81  (2.591) | 2.30  (3.659) | 1.18  (1.535) | 0.151 | 1.913 (2) |
| **WL** |  |  |  |  |  |  |  |  |  |
| Age | 55.96  (12.54) | 56.13  (10.765) | 0.924 | 0.095 (186) | 55.96 (12.540) | 56.48  (10.015) | 55.90  (11.289) | 0.967 | 0.034 (2) |
| Sex | 16/69 | 28/75 | 0.178 | 1.816  (1) | 16/69 | 12/28 | 16/47 | 0.349 | 2.105 (2) |
| Education in years | 13.55  (3.489) | 12.957  (3.138) | 0.258 | -1.136 (160) | 13.550 (3.489) | 12.649  (3.401) | 13.164  (2.961) | 0.403 | 0.913 (2) |
| EQ VAS | 81  (15.958) | 80.50  (16.535) | 0.834 | -0.210 (183) | 81  (15.958) | 82.08  (15.75) | 79.50  (17.06) | 0.726 | 0.321 (2) |
| MARS-5i | 24.54  (0.997) | 24.61  (1.338) | 0.734 | 0.340 (171) | 24.54  (0.997) | 24.45  (1.811) | 24.71  (0.889) | 0.537 | 0.624 (2) |
| BRIEF  (3 item) | 9.75  (1.779) | 9.46  (2.553) | 0.37 | -0.898 (174,92) | 9.75  (1.779) | 8.84  (2.892) | 9.84  (2.27) | 0.065 | 2.774 (2) |
| BDI-9 | 2.60  (2.691) | 3.26  (3.789) | 0.182 | 1.339 (173,27) | 2.6  (2.691) | 3.82  (4.318) | 2.90  (3.403) | 0.182 | 1.724 (2) |
| AUDIT | 1.54  (2.048) | 1.84  (2.979) | 0.48 | 0.708 (150) | 1.54  (2.048) | 2.48  (3.667) | 1.41  (2.353) | 0.141 | 1.983 (2) |

Supplement1. Results in health-related variables: comparisons of evaluating peer support among the groups before the waiting list (preWL) and during the waiting list (WL) period.

|  | **Comparison1.** | | | | **Comparison2.** | | | | | |
| --- | --- | --- | --- | --- | --- | --- | --- | --- | --- | --- |
|  | **RECIEVED1** | **NOT RECEIVED1** | Sig | F/chi (df) | **RECEIVED2** | **WOULD  HAVE REQUESTED2** | **NOT REQUESTED2** | Sig | F/chi (df) | posthoc |
| Age | 56.73 (12.05) | 55.13 (10.15) | 0.369 | -0.90 (192) | 56.73 (12.05) | 56.95 (7.85) | 54.31 (11.02) | 0.474 | 0.748 (2) |  |
| Sex | 33/100 | 13/48 | 0.595 | 0.283 (1) | 33/100 | 6/13 | 7/35 | 0.388 | 1.892 (2) |  |
| Education in years | 13.42 (3.60) | 13.038 (2.74) | 0.458 | -0.744 (130,82) | 13.415 (3.60) | 12.588 (2.15) | 13.250 (2.98) | 0.637 | 0.452 (2) |  |
| QoL VAS | 80.50 (17.16) | 79.58 (17.13) | 0.731 | -0.34  (189) | 80.50 (17.16) | 75.28 (19.96) | 81.43 (15.66) | 0.42 | 0.873 (2) |  |
| MARS-5 | 24.59 (0.95) | 24.54 (1.55) | 0.788 | -0.269 (175) | 24.59 (0.95) | 24 (2.56) | 24.80 (0.56) | **0.049 *** | **3,065 (2)** | NS |
| BRIEF (3 item) | 9.56 (2.09) | 9.64 (2.57) | 0.815 | 0.234 (187) | 9.56 (2.09) | 8.68 (3.48) | 10.10 (1.88) | 0.074 | 2.645 (2) |  |
| BDI-9 | 2.67 (2.72) | 3.47 (4.31) | 0.192 | 1.315 (80,71) | 2.67 (2.72) | 5.56 (5.87) | 2.56 (3.08) | **0.002 *** | **6.618 (2)** | **NS** |
| AUDIT | 1.56 (2.24) | 2.02 (3.23) | 0.307 | 1.025 (154) | 1.56 (2.24) | 3.07 (4.20) | 1.50 (2.54) | 0.09 | 2.444 (2) |  |

Supplement2. Results in health-related variables: comparisons of evaluating peer support among the groups during the ICU period.

|  | **Comparison1.** | | | | **Comparison2.** | | | | | | |
| --- | --- | --- | --- | --- | --- | --- | --- | --- | --- | --- | --- |
|  | **RECIEVED1** | **NOT RECEIVED1** | Sig | F/khi (df) | **RECEIVED2** | **WOULD  HAVE REQUESTED2** | **NOT REQUESTED2** | Sig | F/khi (df) | posthoc |  |
| Age | 56.42 (12.04) | 55.57 (10.18) | 0.638 | -0.472 (191) | 56.42 (12.04) | 56.93 (7.65) | 55.14 (10.91) | 0.788 | 0.239 (2) |  |  |
| Sex | 35/100 | 10/48 | 0.191 | 1.711 (1) | 35/100 | 2/12 | 8/36 | 0.406 | 1.802 (2) |  |  |
| Education  in years | 13.778 (3.52) | 12.24 (2.61) | **0.002*** | **-3,116 (123,97)** | 13.778 (3.52) | 11.923 (2.29) | 12.351 (2.73) | **0.022 *** | **3.912 (2)** | **S** |  |
| QoL VAS | 80.77 (16.81) | 78.60 (17.78) | 0.422 | -0.805 (188) | 80.77 (16.81) | 74.29 (19.10) | 79.98 (17.34) | 0.403 | 0.912 (2) |  |  |
| MARS-5 | 24.60 (0.89) | 24.52 (1.65) | 0.656 | -0.447 (175) | 24.60 (0.89) | 23.93 (2.97) | 24.71 (0.84) | 0.088 | 2.467 (2) |  |  |
| BRIEF  (3 item) | 9.72 (2.06) | 9.32 (2.65) | 0.262 | -1.02 (86,75) | 9.72 (2.06) | 7.79 (3.17) | 9.81 (2.28) | **0.007  *** | **5.128 (2)** | **S** |  |
| BDI-9 | 2.66 (2.84) | 3.56 (4.25) | 0.159 | 1.422 (73,53) | 1.66 (2.49) | 6.57 (6.01) | 2.50 (2.86) | **<0.001 **** | **10.023 (2)** | **NS** |  |
| AUDIT | 1.66 (2.49) | 1.78 (2.77) | 0.798 | 0.256 (154) | 1.66 (2.49) | 4.38 (5.26) | 1.15 (1.20) | **0.005 *** | **5.435 (2)** | **NS** |  |

Supplement3. Results in health-related variables: comparisons of evaluating peer support among the groups during the first year period.
